# Migrants on hemodialysis (HD): clinical characteristics, outcome and quality of life

**DOI:** 10.1007/s40620-025-02281-x

**Published:** 2025-05-06

**Authors:** Gaetano Alfano, Alberto Albinelli, Camilla Ferri, Roberta Romaniello, Oliviero Giuseppe, Ylenia Cancelli, Giulia Ligabue, Silvia Giovanella, Niccolò Morisi, Francesco Fontana, Riccardo Magistroni, Gabriele Donati

**Affiliations:** 1 Nephrology Dialysis and Transplant Unit, University Hospital of Modena, Azienda Ospedaliero - Universitaria di Modena, via del Pozzo, 71, 41124 Modena, Italy; 2 Faculty of Medicine and Surgery, University of Modena and Reggio Emilia, Modena, Italy; 3 Department of Medical and Surgical Sciences, Alma Mater Studiorum - Università di Bologna, Bologna, Italy; 4 Surgical, Medical and Dental Department of Morphological Sciences, Section of Nephrology, University of Modena and Reggio Emilia, Modena, Italy

**Keywords:** Migrant, Refugee, Frail population, Late nephrological referral, End-Stage Kidney Disease (ESKD), Hemodialysis (HD), Chronic Kidney Disease (CKD), Peritoneal Dialysis (PD), Kidney transplantation, Quality of Life (QoL), Vascular access

## Abstract

**Background:**

Migrants who live on hemodialysis (HD) face unique challenges due to social, linguistic and cultural barriers. This study aimed to describe the demographic and clinical profile of migrants on HD, and compare these findings with the national dialysis population.

**Methods:**

A retrospective study was conducted on the HD population at the University Hospital of Modena, Italy. Migrants were defined as adults who entered the country legally or illegally for employment, family reunification and/or to seek asylum.

**Results:**

Migrants accounted for 18.2% (55 patients) of the HD population (302 patients) at our center. This group included individuals who came from Africa (61.8%), Europe (20%), Asia (16.4%), and Latin America (1.8%). About one-third (37.5%) arrived in Italy illegally. Most of the migrants (78.1%) were unaware of their kidney condition upon arrival in Italy. Migrants began dialysis at a younger age compared to Italian HD patients (*P* < 0.001). A higher rate of late referral (*P* < 0.001) and use of temporary vascular access (*P* = 0.015) was observed among migrants. No differences were found in the prevalence of hypertension (*P* = 0.19), diabetes (*P* = 0.27), and cardiovascular comorbidities (*P* = 0.055). Only 34.7% of potentially eligible kidney transplant recipients were evaluated for transplantation. Migrants had a significantly higher total EuroQol 5-Dimension 5-Level (EQ-5D-5L) score index (*P* = 0.046) and reported fewer problems with anxiety/depression (-29.4%; *P* = 0.03).

**Conclusion:**

Migrants started HD at a younger age and had a higher rate of late referral compared to Italian patients. Consequently, dialysis initiation often occurred with temporary vascular access. Despite these issues and limited access to the kidney transplant waiting list, migrants overall reported a better quality of life.

**Graphical abstract:**

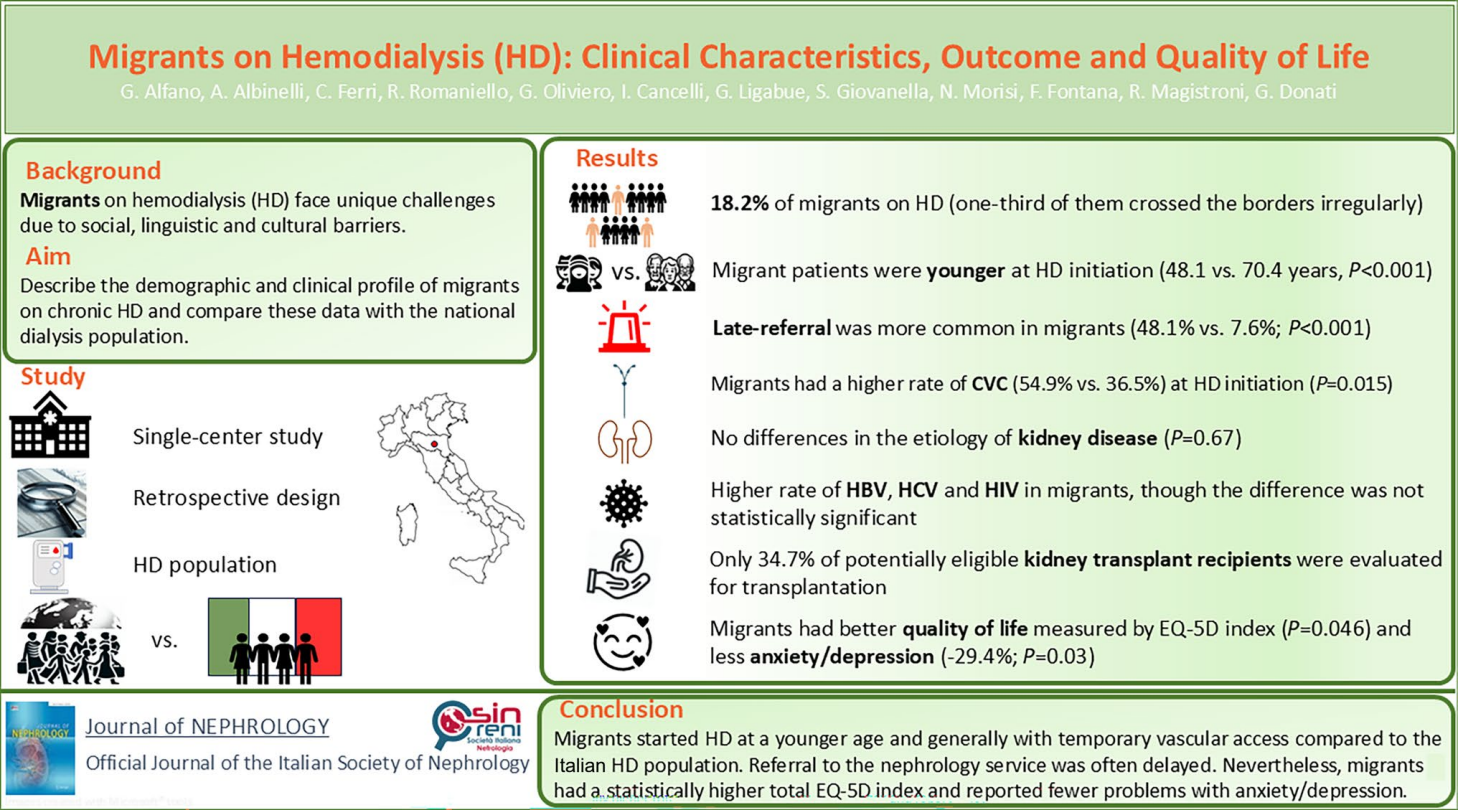

**Supplementary Information:**

The online version contains supplementary material available at 10.1007/s40620-025-02281-x.

## Introduction

Since the end of the Second World War, Europe has been a crossroads of human mobility. The combination of relative economic prosperity and political stability of European (EU) countries has exerted a considerable pull effect on non-citizen workers, whereas the multiple conflicts in neighboring countries have attracted the migratory flow towards EU countries capable of providing protection and assistance to refugees and asylum-seekers [1]. The most recent epidemiological data documented that 23.7 million citizens of non-member countries live in Europe, representing 5.3% of the population. Italy along with Germany, Spain and France host 70.3% of the total number of non-nationals living in all EU member states [2]. For the healthcare community, caring for these people is particularly challenging. This is especially true for patients affected by chronic conditions such as chronic kidney disease (CKD). The increasing number of people with end-stage kidney disease (ESKD) who move away from their birth place or usual place of residence (denoted as migrants in this text) [1] increases the demand for nephrological care compared to the national dialysis population. These patients are often unaware of being severely affected by ESKD and lack a pre-dialysis follow-up. Referral to nephrologists is frequently delayed, resulting in crash landing presentation requiring urgent and unplanned hemodialysis (HD) start. Typically, this condition triggers the start of a series of time-consuming administrative procedures to ensure a lifesaving treatment for patients temporarily not covered by free healthcare assistance.

Further limitations make the management of these patients challenging. Cultural background may contribute to overlooking severe clinical conditions, and linguistic barriers often limit the possibility of understanding prescribed pharmacological therapy thereby jeopardizing access to the kidney transplant waiting list. Additionally, the absence of a stable residence and inadequate hygienic conditions make the option of peritoneal dialysis (PD) or home hemodialysis (HHD) less viable for this population.

In this context, the engagement of the dialysis staff is especially valuable. In the process of embodying the migrant’s cultural and social differences, they ensure a holistic approach to dialysis treatment prioritizing equitable dialysis treatment and access to the kidney transplant list. Nevertheless, the contact between individual healthcare workers and migrants is not straightforward and reflects the substantial challenges encountered in the process of socio-cultural and economic integration seen at the macro-social level. As a result, the dialysis staff, involved in the multiple activities of the dialysis routine, are often not familiar with the clinical characteristics, personal experience, and expectations of these patients. Our study stems from the need to describe the demographic and clinical profile of migrants on chronic maintenance HD and to compare them with the national dialysis population in a healthcare system based on the principles of universality and equity.

## Materials and methods

### Study design

An observational retrospective study was conducted on the HD population at the University Hospital (AOU) of Modena, Italy. All HD patients with ESKD undergoing chronic HD > 3 months and aged > 18 years from December 1, 2021, to August 31, 2022, were enrolled in the study.

### Population

Patients were subsequently categorized based on their origin into “migrants” and “non-migrants.”

A migrant was defined as an individual who moves away from their usual place of birth or residence, either within a country or across an international border, temporarily or permanently, for various reasons. Given the absence of a universally accepted definition of migrant and its precise legal connotation, in this study, the term migrant refers to individuals born outside Italy who came to our country later in life for reasons related to employment, family reunification, economic or political factors. The study included undocumented people who entered illegally, and excluded individuals born abroad whose parents were Italian nationals living in Italy. For simplicity, we use the terms migrants and refugees essentially interchangeably. The term “non-migrant population” refers to individuals either born in Italy or born abroad but who spent their childhood in Italy under the custody of parents or tutors.

### Healthcare system

The Italian National Health Service (SSN) was created in 1978 after the failure of a social health insurance system established in 1970, which left around 7% of the population without coverage. It was created on the principle that healthcare is a fundamental human right, available to all citizens regardless of their ability to pay. While private healthcare still exists, it is primarily for those willing to pay for additional services or treatments not covered by the SSN, such as dentistry or psychological care.

In Italy, urgent healthcare is provided to everyone, including undocumented individuals. Asylum seekers and refugees can access healthcare services on the same terms as Italian citizens. To access these services, individuals must register with the Italian public healthcare system. Once registered, a health card grants access to various services, including a family doctor, specialist medical visits, blood tests, vaccinations, hospitalization, and other health treatments depending on specific needs. For undocumented individuals, non-urgent healthcare services are generally provided on a private, out-of-pocket basis. However, when requesting care, individuals can be assigned an identification code known as an STP (temporarily present foreigner), which is valid for six months and renewable. If the foreign citizen does not have sufficient funds, they will only be required to pay a portion of the quota (participation fee). In cases where the individual is entirely without funds (i.e., in a state of indigence), they can be exempted from paying the participation fee by submitting a “declaration of indigence”.

### Data collection

Data were collected from the patient's HD records, the regional kidney replacement therapy (KRT) registry and other electronic health records. Some other data were gathered following an interview with HD patients.

Late nephrological referral is defined as less than three months between the first nephrology evaluation and the initiation of KRT. In this study, late referral is set at less than three months. Patients were also asked to fill in a specific Quality of Life (QoL) questionnaire after obtaining informed consent. The questionnaire we used was the “EQ-5D,” (authorization ID 42254), which consists of five dimensions (5D) that capture how patients perceive their overall QoL. The domains assessed include mobility, self-care, usual activities, pain/discomfort, anxiety/depression with 5 levels (5L) of problem severity in the responses. A second questionnaire, called the EQ Visual Analogue Scale (VAS), was also administered to patients. This tool uses a visual analogue scale ranging from 0 to 100, where 0 represents the worst health imaginable and 100 represents the best health imaginable of their ‘health today’. The EQ-5D-5L Italian value provided by EuroQol [3] on August 7, 2024 was used for the calculation of the EQ-5D-5L indices. EQ-5D-5L index scores range from 0.59 to 1, where 1 is the best possible health state.

The study was approved by the AVEN ethical committee of the Emilia Romagna Region (507/2021/OSS/AOUMO SIRER ID 2845).

### Statistical analysis

After verifying the data distribution, continuous variables were presented as median and interquartile range, while categorical variables were expressed as frequency and/or percentage.

Differences between the two groups with non-normal distribution were assessed using the Mann–Whitney U test, while differences in categorical data were evaluated using the chi-square test or Fisher’s exact test (for sample sizes < 15) as appropriate. *P* values less than 0.05 were considered statistically significant. IBM SPSS^®^ Statistics 23 (SPSS Inc., Armonk, NY, USA) and Stata 14.1 (StataCorp, College Station, TX) were used for all analyses.

## Results

### Total hemodialysis population

The total population included 302 prevalent patients undergoing chronic HD. This population was subdivided into migrants (*n* = 55; 18.2%) and non-migrants (*n* = 247; 81.7%). Overall, the median age of enrolled patients was 70.5 years (IQR 56.8–79.8) and the majority were male (61.9%).

HD treatment began at a median age of 67.6 years (IQR 52.4–77) and dialysis vintage was estimated to be 2.6 years (IQR 1–5.2). As depicted in Table 1, glomerular diseases (*n* = 62; 20.5%), hypertensive nephroangiosclerosis (*n* = 60; 20.1%) and diabetes (*n* = 49; 16.2%) represented the most common causes of ESKD. Concerning vascular access for HD at the time of enrollment, native arteriovenous fistula (AVF) accounted for 52.9%, followed by tunneled internal jugular central venous catheter (CVC) (46%) and prosthetic AVF (0.9%).

**Table 1:** Demographic and clinical characteristics of migrant and non-migrant patients on hemodialysis

	All patients	Migrant	Non-migrant	*P* value
	*N* = 302 (100%)	*N* = 55 (18.2%)	*N* = 247 (81.7%)	
Age at HD initiation (y)	67.6 (52.4–77)	48.1 (39.7–56.7)	70.4 (59.1–78.9)	** < 0.001**
Male sex, n. (%)	187 (61.9)	38 (69)	149 (60.3)	0.21
Comorbidity, n. (%)				
Hypertension	210 (69.5%)	41 (74.5)	169 (68.4)	0.19
Diabetes mellitus	107 (35.4)	16 (29.1)	91 (36.8)	0.27
Cardiovascular disease	156 (51.6)	22 (40)	134 (54.2)	0.055
HBV	8 (2.6)	4 (7.2)	6 (1.6)	0.06
HCV	9 (2.9)	3 (5.4)	6 (2.4)	0.23
HIV	4 (1.3)	2 (3.6)	2 (0.8%)	0.09
Etiology of ESKD, n. (%)				0.67
Glomerular disease	62 (20.5)	14 (25)	48 (19.4)	
Hypertensive nephroangiosclerosis	60 (20.1)	14 (25)	46 (18.6)	
Diabetes	49 (16.2)	12 (21.8)	37 (14.9)	
Interstitial nephritis	27 (10.8)	0 (0)	27 (10.9)	
Genetic	25 (8.2)	3 (5.4)	22 (8.9)	
Other	25 (8.2)	4 (7.2)	21 (8.5)	
Unknown origin	54 (17.8)	8 (15.6)	46 (18.6)	
Late nephrological referral, n. (%)	43 (17.4)	24 (44.4)	17 (7.6)	** < 0.001**
First vascular access for dialysis, n. (%)				**0.015**
Native AVF	171 (56.6)	23 (45.1)	148 (63.5)	
CVC	113 (37.4)	28 (54.9)	85 (36.5)	
QoL assessed by EQ-5D-5L				
EQ-5D-5L index value	0.95 (0.85–1)	1 (0.95–1)	0.94 (0.81–1)	**0.046**
EQ-VAS	90 (75–95)	90 (80–92)	87.5 (71.2–95)	0.62

HD, hemodialysis; HBV, Hepatitis B Virus; HCV, Hepatitis C Virus; HIV, Human Immunodeficiency Virus; ESKD, End-Stage Kidney Disease; AVF, Arteriovenous Fistula; CVC, Central Venous Catheter; QoL, Quality of Life; EQ-5D-5L, EuroQoL 5-Dimension 5-Level; EQ-VAS, EuroQoL Visual Analog Scale

Median EQ-5D-5L index value and EQ-VAS were 0.95 (0.85–1) and 90 (75–95), respectively. In this cohort, problems (slight, moderate, severe or extreme severity) were self-reported by 58.3% of the patients. In particular, problems included the domains of mobility (28.3%), self-care (25%), usual activities (50%), pain/discomfort (31.6%) and anxiety/depression (31.6%) (Table 1).

### Demographic and clinical characteristics of the migrant population

**Fig. 1:**
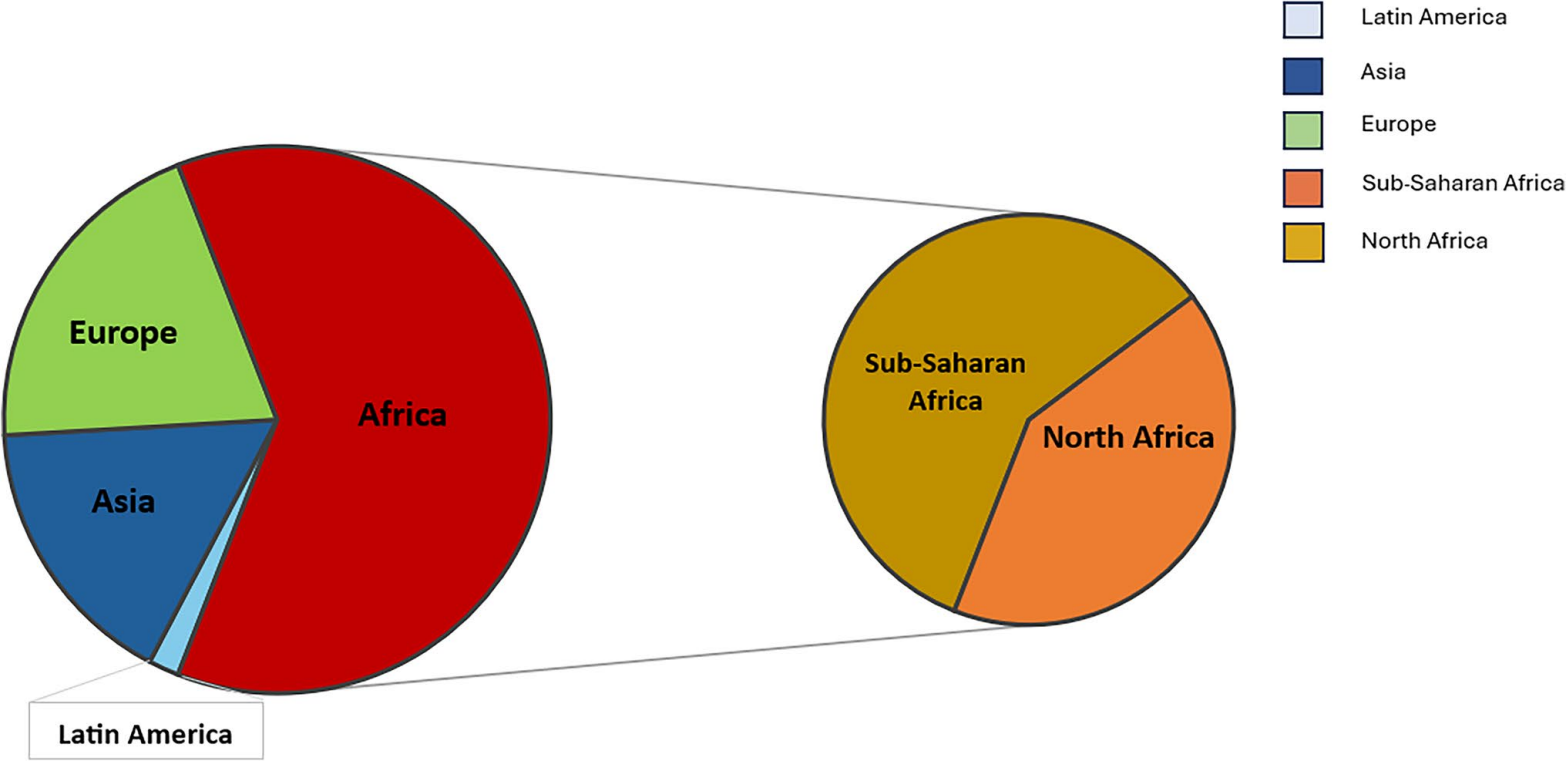
Migrant's continent of origin

As shown in Fig. 1, 55 migrants moved to Italy principally from Africa (*n* = 34; 61.8%), Europe (*n* = 11; 20%), Asia (*n* = 9; 16.4%) and Latin America (*n* = 1; 1.8%). The African population originated from Sub-Saharan Africa (61.8%) and from North Africa (38.2%), whereas 100% of Europeans moved from Eastern Europe. The Gross National Income (GNI) per capita was used as an economic indicator to assess the conditions of migrants’ home countries at the time of their departure. Overall, 24% of migrants moved from low-income countries, 58% from lower-middle-income countries, and 18% from upper-middle-income countries (Supplementary Fig. 1).

The median age of enrolled patients was 53 years (IQR 46.3–60.9) and 69% of them were males. HD was started at a median age of 48.1 years (IQR 39.7–56.7). Migrants arrived in Italy at the age of 33.5 years (IQR 28.3–41.6) and began dialysis treatment 12.3 years later (IQR 6.5–20). Only one patient (1.8%) was already on maintenance HD upon arrival in Italy.

**Fig. 2:**
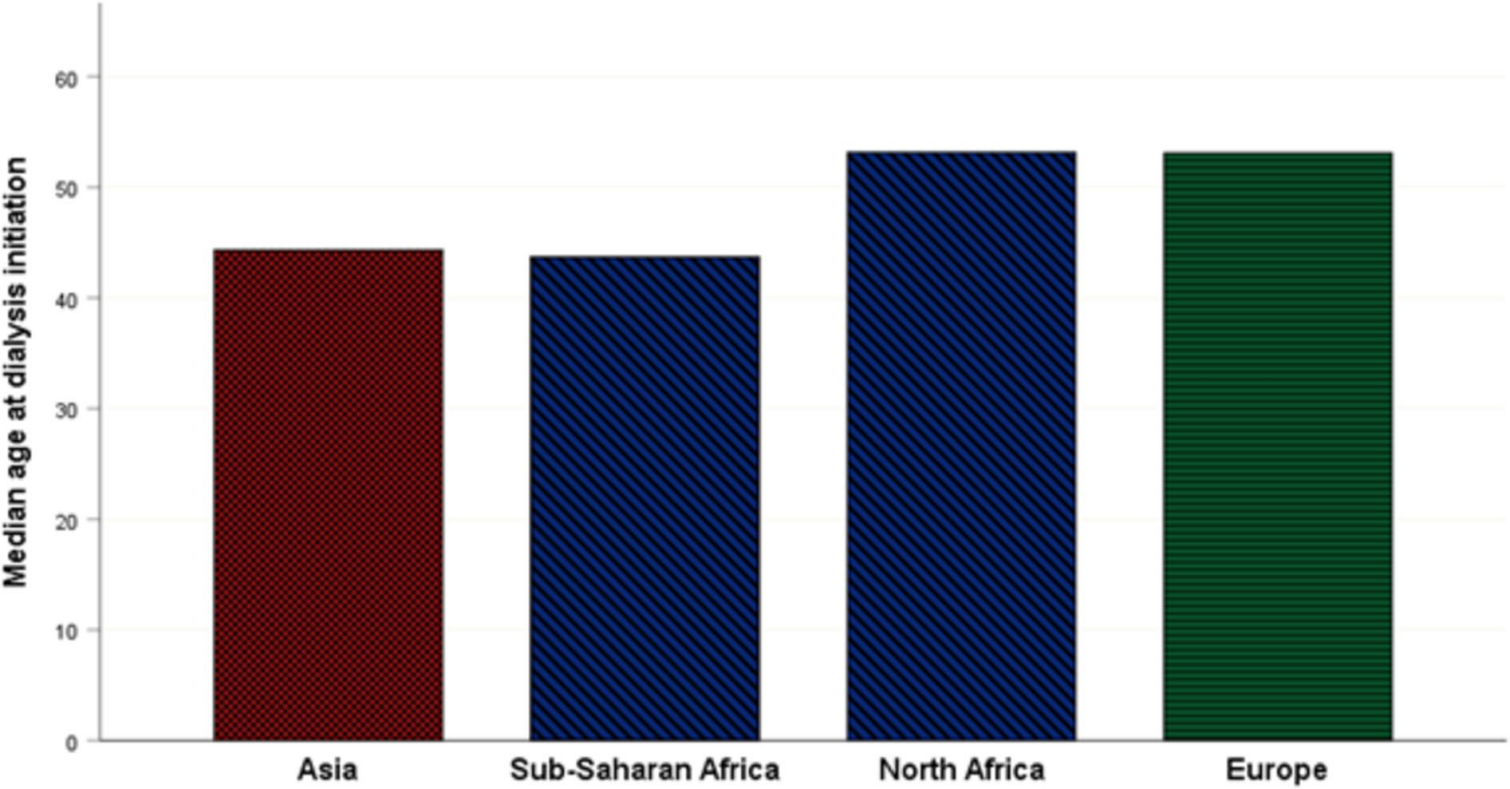
Median age of migrants at hemodialysis initiation

Median age at the start of dialysis was lower for sub-Saharan African (43.6 years [38.7–48.3]) and Asian (44.3 years [39.3–52.1]) patients compared to European (53.1 years [45.7–57.2]) and North African (54.1 years [43.2–67.5]) patients. The difference between the 4 groups was not statistically significant (P = 0.067) (Fig. 2).

Hypertension, diabetes and cardiovascular comorbidities were present in 74.5%, 29.1% and 40% of patients, respectively. Active HBV (i.e., HBsAg positive), HCV and HIV infection were found in 7.2%, 5.4% and 3.6% of the migrant population, respectively.

Despite the relatively young age of the migrants at the start of dialysis compared to non-migrants, kidney biopsy was performed in only 56.3% of cases. Etiology of ESKD mainly involved glomerulonephritis (25%), hypertensive nephroangiosclerosis (25%), diabetic nephropathy (21.9%), and unknown origin (15.6%).

Late referral and first vascular access for dialysis were evaluated in 54 patients as one was already on dialysis upon arrival in Italy. Late referral was common in this population, occurring in 24 out of 54 patients (44.4%). Five of these patients were classified as lost to nephrological follow-up as they underwent the last visit 3.6 (IQR 2.5–5.7) years earlier. CVC was the principal vascular access at dialysis initiation for 28 migrants (43.6% jugular CVC and 7.2% femoral CVC). Twenty-three (45.1%) started with a native AVF and only three (5.4%) started with PD. At the time of the interview, the rate of CVC had decreased to 25.5% and the rate of native AVF had risen to 72.7%; 1.8% of patients had a prosthetic fistula.

After considering absolute contraindications to kidney transplantation eligibility (e.g., obesity, social condition, active pathology, advanced atherosclerosis and the arbitrary age limit of 75), potentially 46 out of 55 migrants could have been eligible for a kidney transplant. At the end of the study, only 16 patients (34.7%) were evaluated for kidney transplant eligibility. Their status on the kidney transplant waiting list was: six were on the active list; four were suspended for clinical reasons; three were under evaluation; three were ineligible.

### Socioeconomic characteristics and quality of life

Due to the complete language barrier affecting the comprehension of questions and the reliability of responses, personal information was retrieved from only 32 patients (58.1%), Consequently, data concerning socioeconomic characteristics and QoL reported below were based on this smaller subgroup of patients. The median age of these patients was 51.7 years (IQR 46.9–59.4) and the predominant gender was male (71.8%). The educational levels indicated that 83.9% had either no formal education (25.8%) or had completed lower secondary education (58.1%), while 12.9% had completed higher secondary education, and 6.5% held a university degree (Supplementary Fig. 1).

The Italian border was crossed legally by migrants with regular documentation (62.5%) and illegally (37.5%) via the Libyan and Balkan routes. Upon their arrival in Italy, the migrants moved on to the Italian territory in search of better social and employment opportunities. Only 11 migrants (34.3%) chose our city as their permanent residence upon arrival in Italy. The primary reasons for migrating to Italy were employment opportunities (81.2%), family reunification (15.7%), and war in Ukraine (3.1%). Among migrants pursuing employment, only 15.3% had a work visa upon arrival in Italy. Furthermore, 38.4% and 15.3% had a first- and/or second-degree relative waiting for them upon their arrival in Italy, respectively. All migrants seeking family reunification and only one war refugee had a first-degree relative in Italy. At enrollment, 19 patients (59.3%) were living in Italy without their families, and 12 of them (63.1%) had first-degree family members residing in their home country.

Regarding employment, the first job in Italy was obtained after 0.93 years (IQR 0–1.01) from arrival, and 21.9% of jobs involved unregistered employment. Despite all patients without language barriers being of working age, only 8 (25%) of them were employed (Supplementary Fig. 1). Notably, one had full-time employment whereas the remaining seven worked part-time. All migrant patients received or were in the process of applying for a monthly disability annuity provided by the Italian welfare system due to advanced kidney failure.

Most of the migrants (78.1%) were unaware of their kidney condition upon arrival in Italy and only a small percentage (14.5%) remembered having received information on PD and/or home HD.

Migrants had a median EQ-5D-5L index value of 1 (0.95–1) and EQ-VAS of 90 (80–92). Problems (slight, moderate, severe or extreme severity) were self-reported in 42% of the patients. In particular, problems included the domains of mobility (15.7%), self-care (10%), usual activities (31.5%), pain/discomfort (10.5%) and anxiety/depression (10%) **(**Table 1**).**

### Comparison between migrant and non-migrant hemodialysis populations

**Fig. 3:**
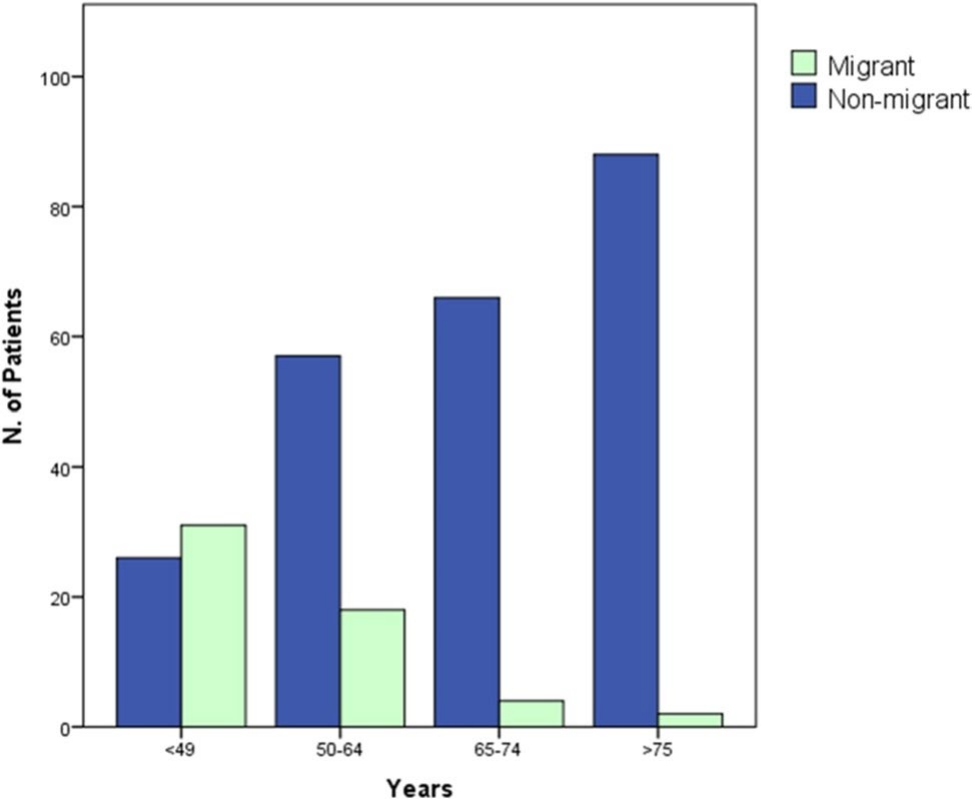
Age groups of patients starting hemodialysis

As depicted in Fig. 3, age at HD start differed between migrant and non-migrant groups. Migrant patients were younger at dialysis initiation (48.1 vs. 70.4 years, *P* < 0.001) and study enrollment (53 vs. 74.1 years, *P* < 0.001). There were no significant differences between the two groups regarding gender (*P* = 0.21) and etiology of CKD (*P* = 0.67). No differences were found in the rates of hypertension (74.5% vs 68.4%, *P* = 0.19), diabetes (29% vs 36.8%; *P* = 0.27) and cardiovascular comorbidities (40% vs 54.2%; *P* = 0.055).

Late referral was more common in migrants (48.1% vs. 7.6%; *P* < 0.001). At HD initiation, the type of vascular access also differed (*P* = 0.015). Migrants had a higher prevalence of CVC (54.9% vs. 36.5%) and a lower prevalence of native AVF (45.1% vs. 63.5%) (*P* = 0.015). Although migrants had a higher percentage of infectious disease, there were no statistically significant differences between migrant and non-migrant groups in terms of HBV (7.2% vs. 2.4%; *P* = 0.089), HCV (5.4% vs. 2.4%; *P* = 0.21) and HIV infection (3.2% vs. 0.8%; *P* = 0.15). Lastly, migrants were less frequently evaluated for kidney transplantation compared to Italian HD patients (34.7% vs 46% (*P* = 0.21).

### Quality of Life

Migrants showed a statistically significant difference in higher total EQ-5D index compared to non-migrants (1 vs 0.94; *P* = 0.046). Migrants experienced fewer problems in mobility (– 16.7%; *P* = 0.22), selfcare (– 19.4%; *P* = 0.1), usual activities (– 25.9%; *P* = 0.09), pain/discomfort (– 19.2%; *P* = 0.21) and anxiety/depression (– 29.4%; *P* = 0.03). EQ-VAS was lower in the migrant group than in the non-migrant group (90 vs 87.5), though the difference did not reach statistical significance (*P* = 0.62).

## Discussion

Management of migrants with ESKD on dialysis presents unique challenges to the nephrological community due to a series of social, linguistic and cultural barriers within this population. These challenges are further compounded by the scarcity of data hindering a comprehensive understanding of the problem. Moreover, despite the efforts of the dialysis staff and allocation of additional resources, there is the perception that the quality of care of these patients is suboptimal and outcomes are poorer compared to the national dialysis population [4–6].

In the present study, we collected data on 55 migrants undergoing chronic HD at a large dialysis center in northern Italy. Birthplaces were Africa (61.8%), Eastern Europe (16.4%), South Asia (16.3%) and Latin America (1.8%). The presence of migrants at our center was well-represented and they accounted for about one-fifth of the overall HD population. These data differ slightly from the results of a recent international survey promoted by the International Society of Nephrology (ISN) reporting a lower prevalence of migrants across dialysis centers. Migrants constituted 1.5% of the dialysis population and only a limited number of centers provided care to more than 20 subjects [7]. It is worth noting that the flow of migrants can vary over time and mainly depends on the proximity to countries experiencing economic crises or armed conflicts [8–10]. Our dialysis center, being located in an industrial area, was the final destination of individuals who mostly sought better employment opportunities (81.2%).

Widening the view on the demographics of this region, the prevalence of migrants on HD treatment is slightly overrepresented with respect to the overall prevalence of migrants in our city. According to the most recent epidemiological study, the prevalence of migrants treated by dialysis was higher than the estimated prevalence of all migrants living in our city (18.2% vs. 13.6%) [11]. Another significant disproportion was observed between the rate of migrants with ESKD on HD compared to those receiving pre-dialysis care. The rate of migrants receiving HD was considerably higher than that of individuals with an estimated glomerular filtration rate (eGFR) < 20 ml/min followed in our pre-dialysis program (18.2% vs. 7.5% [data not published]). These data underline that a consistent number of migrants, unaware of their clinical condition, do not receive adequate pre-dialysis care and remain unprepared for KRT initiation. As a result, migrants had a six-fold higher rate of late referrals compared to the non-migrant population. Our data are confirmed by Astor et al.[12], who reported that late nephrological referral is more common among ethnic minorities. Delay in seeking nephrological care exposes patients to rapid progression of kidney disease and is associated with a poorer clinical state at the start of KRT due to the lack of nephrological care and permanent vascular access [13]. Unplanned dialysis start also compromises the ability of the patient to make an informed choice of dialysis modality [14]. Indeed, only a small percentage of our migrant patients (14.5%) declared to have received education on the different dialysis modalities, including home dialysis. Likewise, late referral also precludes the timely placement of permanent vascular access [13, 15]. This is almost invariably associated with crash dialysis start requiring hospitalization and the placement of a temporary CVC, a solution that inevitably occurred in 54.9% of our migrant population.

A comparative analysis of the age of both migrant and non-migrant groups revealed significant differences between these cohorts. Migrants began treatment at a younger age compared to Italian patients (48.1 vs. 70.4 years). Specifically, Sub-Saharan African patients, who represent the majority of migrants [*n*. = 21], were the youngest group (43.6 years) to commence HD. These data align with a recent study conducted in Spain where the authors found an almost 20-year difference in age at dialysis initiation (57.1 vs. 72.5 years) between the two populations [16]. Similar results have been reported in a multiethnic population in Australia where the median age of the adult East/South-East Asian and Pacific Islander immigrant populations was lower than that of adult Australian-born non-indigenous people [17].

The etiology of CKD between the two groups was similar. According to data extrapolated from Germany [18] and from the French national register [19], one fifth of our migrant HD patients had an undetermined kidney diagnosis of ESKD. However, if we consider that the hypothesized hypertensive nephropathy is the final manifestation of a different underlying primary disorder, and that only half of the migrants have a biopsy-proven diagnosis of kidney disease, the prevalence of unknown kidney disease is likely underestimated in our study. The early development of ESKD in this subset of patients, often without any precise knowledge of the underlying disease, raises many questions about the current practices for screening for kidney disease in both their birth and destination countries. Migrants are a vulnerable population, often originating from countries with limited access to healthcare services, which might fail to detect potentially preventable kidney diseases during childhood. Indeed, approximately three-quarters of the migrants were unaware of their kidney condition upon arrival in Italy. Once documented and undocumented migrants arrive in Italy, they are assisted by the Italian Health System. After receiving initial emergency care, undocumented patients can undergo a first basic health evaluation. Depending on age and country of origin, migrants are usually screened for infectious diseases, diabetes, cervical cancer. Kidney disease screening usually occurs during a second evaluation phase as part of secondary prevention efforts, which include active search for diseases, even in subclinical forms. Migrants with regular residency status are assigned a family physician, similar to Italian citizens [20]. Nevertheless, the aforementioned inherent limitations of this population, along with the lack of knowledge about the rules for accessing healthcare, create sometimes insurmountable obstacles preventing migrants from seeking medical assistance. However, it is worth noting that the diagnosis of ESKD or other chronic diseases in Italy does not preclude migrants from obtaining permanent residency.

The differences in the rate of hypertension, diabetes and cardiovascular diseases were not statistically significant between the two groups of migrant and non migrant patients on HD. The prevalence of hepatitis and HIV was higher in migrants, though without reaching statistical significance. The absence of HBV vaccination programs in their birth countries increases the risk of community transmission of the virus, which is particularly concerning within dialysis units. Regarding of HIV, the frequent movement of migrants in search of better living conditions, combined with the fear of discrimination, complicates the monitoring of therapy adherence and viral load. Early screening and education to maintain close follow-up is extremely important, as untreated HIV, especially in individuals of African descent [21], significantly increases the risk of developing ESKD [22].

Evaluation of the status of immigrants on the transplant list shows that approximately two-thirds of migrant patients, though potentially eligible, are neither on the list nor are being assessed for kidney transplantation eligibility. However, it is worth noting that in Italy, kidney transplantation is actively promoted and migrant status itself does not constitute a barrier. Yet, multiple non-financial obstacles can significantly hinder access to transplantation. Language barriers, limited health literacy, and cultural differences often result in poor engagement with the healthcare system and a lack of awareness about transplantation as a treatment option. Migrants may struggle to navigate the complex transplant pathway, frequently leading to missed appointments. Fear of job loss represents another critical barrier, as pre-transplant evaluations require multiple medical visits, which may be challenging for individuals with precarious employment. Moreover, migrants often have limited self-advocacy in healthcare and exert less pressure on physicians to prioritize their placement on the waiting list. Additionally, frequent movement between cities and travel to their home country may further disrupt continuity of care.

Similar experiences are widely reported. A British study by Rudge et al. [23] indicates that ethnic minorities undergo dialysis longer and are less likely to be wait-listed for deceased-donor transplants, resulting in longer wait times and reduced transplantation likelihood. Patzer et al. [24, 25] noted that Black patients have lower access to steps leading to transplants, including evaluation and listing. Scientific literature documented that younger Black patients on dialysis have a double risk of death compared to their White counterparts, even after adjusting for many demographic factors and comorbidities [26]. Moreover, a long amount of time spent on dialysis is associated with graft failure and mortality after kidney transplantation [27].

An important finding in our study concerns QoL. A significant difference was observed in scores on the QL-5D questionnaire and migrants reported a better overall QoL. This group performed particularly well in the anxiety/depression domain of the QL-5D questionnaire, showing 30% fewer problems compared to Italian patients. Few studies explored the QoL of migrants receiving dialysis. As a general consensus, migrants are highly susceptible to developing mood dysfunction due to the numerous disadvantages they face. Nevertheless, migrants can show better mental health [28] probably due to the social resilience that is embodied in their culture [29]. Patel et al. [30] found that greater spirituality and religiosity among African Americans correlate with higher perceived social support and QoL, and lower negative perceptions of illness and depression, acting as coping mechanisms for ESKD patients. However, due to multiple factors, this result should be interpreted cautiously. One potential confounder was the difference in age between the migrants who completed the questionnaire. Older national patients tended to experience declines in health-related QoL, particularly those with multiple comorbidities, which led to limited functional status compared to younger patients. Additionally, the questionnaire was filled in by a subgroup of migrants (*n* = 32 vs. *n* = 55) with no linguistic barriers. This subgroup likely represents migrants with better adaptation to a new cultural context and increased participation in society and health services. It is possible that migrants experiencing social marginalization due to language barriers could face greater difficulties navigating a healthcare system that is expected to be used autonomously. This unintentional selection bias may underestimate the prevalence of anxiety or depression symptoms, which might mitigate the positive results observed in our study.

This study has several other limitations that should be acknowledged. First, the small sample size limited our ability to detect independent relationships between clinically significant variables between migrant and non-migrant populations. Second, the demographic differences, especially in terms of age, may have influenced the results of the study. Third, the small number of migrants did not allow us to subdivide them into documented and undocumented patients. Finally, the retrospective analysis of these data could be subject to potential data collection biases, and the single-center design may not fully reflect the national context. However, these findings highlight an emerging issue for nephrologists, as the migrant population faces greater challenges compared to the native population.

## Conclusion

This study sheds light on significant differences between migrant and non-migrant populations receiving HD within a healthcare system based on universality and equity. Migrants tend to initiate HD at a younger age and often experience late nephrological referral leading to crash dialysis starting with temporary vascular access. More importantly, access to the kidney transplant waiting list seems to be limited despite the absence of institutional or economic barriers that may disproportionately affect this population. Nevertheless, our data suggest a better QoL among migrants, a result that needs confirmation on a larger scale as it may be biased due to demographic differences between the groups and a selection bias in case of migrants who completed the EQ-5D questionnaire. Further interventions ranging from universal screening for CKD to expedited registration on the kidney transplant waiting list might positively influence the healthcare experience of this vulnerable population.

## Supplementary Information

Below is the link to the electronic supplementary material.

Supplementary Figure 1. Socioeconomic condition and education level of migrants. Supplementary file1 (JPG 93 KB)

## Data Availability

The study protocol was approved by the Regional Ethical Committee (507/2021/OSS/AOUMO SIRER ID 2845).

## Abbreviations

APD: Automatic Peritoneal Dialysis
AVF: Arteriovenous Fistula
CAPD: Continuous Ambulatory Peritoneal Dialysis
CKD: Chronic Kidney Disease
CVC: Central Venous Catheter
PD: Peritoneal Dialysis
ESKD: End-Stage Kidney Disease
EQ-5D-5L: EuroQol 5-Dimension 5-Level (Quality of Life Assessment)
EQ-VAS: EQ Visual Analogue Scale
HD: Hemodialysis
HIV: Human Immunodeficiency Virus
HBV: Hepatitis B Virus
HCV: Hepatitis C Virus
HHD: Home Hemodialysis
