# Supplementary figures and images for: Migrants on hemodialysis (HD): clinical characteristics, outcome and quality of life
Source: J Nephrol. 2025 May 6;38(3):1057–67. doi: 10.1007/s40620-025-02281-x (PMC12166013; doi:10.1007/s40620-025-02281-x)

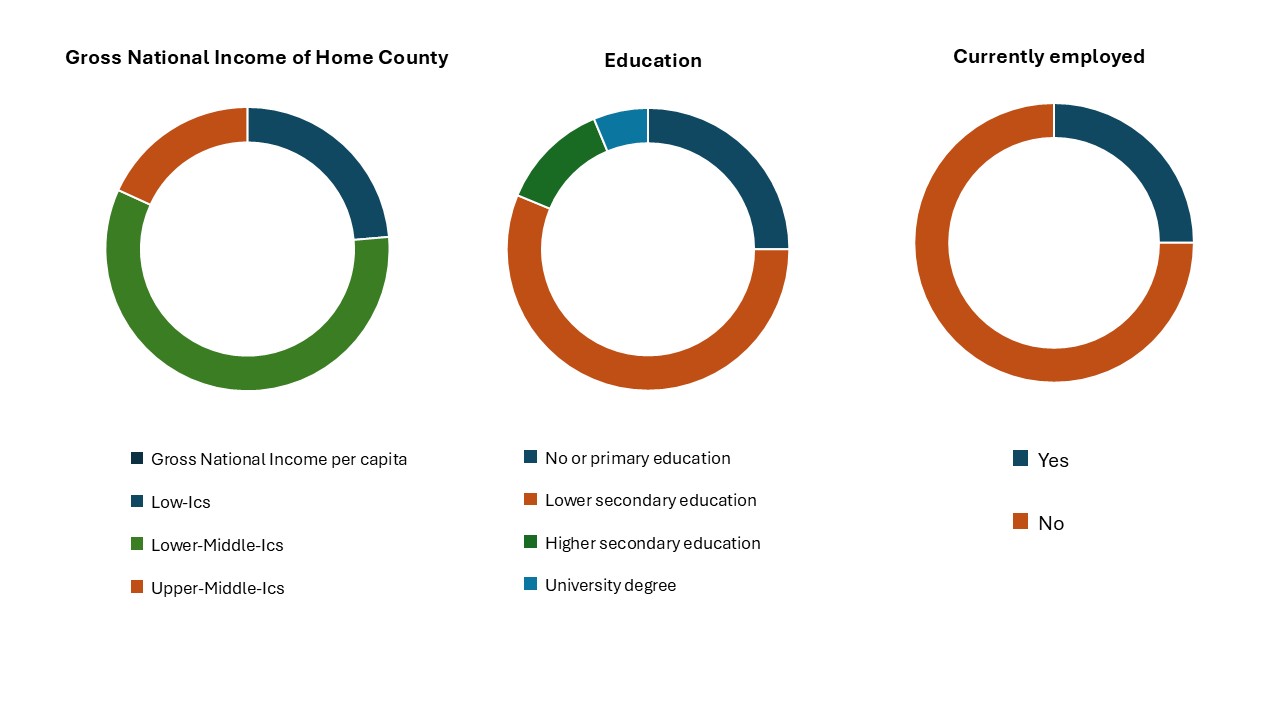

Supplement: Supplementary file 1 — Supplementary Figure 1. Socioeconomic condition and education level of migrants. Supplementary file1 (JPG 93 KB) [file 40620_2025_2281_MOESM1_ESM.jpg]
